# Crystal structure of 1,3-bis­(1,3-dioxoisoindolin-1-yl)urea dihydrate: a urea-based anion receptor

**DOI:** 10.1107/S1600536814022144

**Published:** 2014-10-24

**Authors:** Felipe Medrano, Sergio Lujano, Carolina Godoy-Alcántar, Hugo Tlahuext

**Affiliations:** a Centro de Investigaciones Químicas, Universidad Autónoma del Estado de Morelos, Av. Universidad 1001 Col., Chamilpa, CP 62209, Cuernavaca, Mexico

**Keywords:** crystal structure, isoindoline, urea, phthalimides, protection of primary amines, urea-based anion receptor

## Abstract

The whole mol­ecule of the title compound, C_17_H_10_N_4_O_5_·2H_2_O, is generated by twofold rotation symmetry and it crystallized as a dihydrate. The planes of the phthalimide moieties and the urea unit are almost normal to one another, with a dihedral angle of 78.62 (9)°. In the crystal, mol­ecules are linked by N—H⋯O and O—H⋯O hydrogen bonds, forming a three-dimensional framework structure. The crystal packing also features C—H⋯O hydrogen bonds and slipped parallel π–π inter­actions [centroid–centroid distance = 3.6746 (15) Å] involving the benzene rings of neighbouring phthalimide moieties.

## Abstract 2

The title compound possesses twofold rotation symmetry, with the planes of the phthalimide moieties inclined to one another by 73.53 (7)° and by 78.62 (9)° to that of the urea unit. In the crystal, mol­ecules are linked *via* N—H⋯O and O—H⋯O hydrogen bonds, forming a three-dimensional framework structure.

## Chemical context   

Hydrogen bonding and π–π inter­actions are two of the principal forces which determine structure, self-assembly and recognition in some chemical and biological systems (Lehn, 1990 ▶). A variety of urea-based anion receptors of varying complexity and sophistication have been synthesised (Amendola *et al.*, 2010 ▶). It has been shown that the efficiency of urea as a receptor subunit depends on the presence of two proximate polarised N—H fragments, capable of (i) chelating a spherical anion or (ii) donating two parallel hydrogen bonds to the O atoms of a carboxyl­ate or of an inorganic oxoanion. A review of the biological activity of phthalimides has been published by Sharma *et al.* (2010 ▶) and a review of its the supra­molecular chemistry by Barooah & Baruah (2007 ▶). Phthalimides and isoindolines have been shown to possess photophysical properties and have applications as colourimetric and other types of anion sensors (Griesbeck & Schieffer, 2003 ▶; Griesbeck *et al.*, 2007 ▶, 2010 ▶; Devaraj & Kandaswamy, 2013 ▶). In our ongoing research on 1,3-dioxoisoindolines as anion receptors (Lujano, 2012 ▶), we report herein on the synthesis and crystal structure of the title urea-based anion receptor.
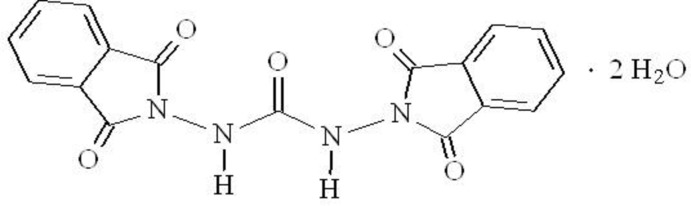


## Structural commentary   

**Figure 1:**
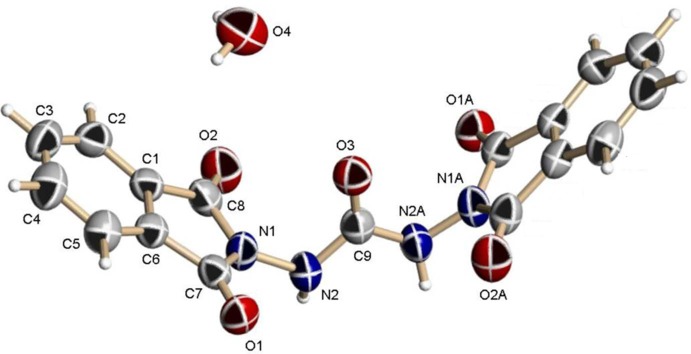
The mol­ecular structure of the title mol­ecule, showing the atom labelling. Displacement ellipsoids are drawn at the 50% probability level. Atoms with the suffix A are generated by the symmetry operator (−*x*, *y*, −*z* + 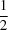) and the symmetry-related water mol­ecule is not shown.

The mol­ecular structure of the title compound is illustrated in Fig. 1 ▶. The mol­ecule is located on a crystallographic twofold rotation axis that bis­ects the central C9=O3 bond. The planes of the phthalimide unit (N1/C1–C8) and the urea unit [N2—C9(=O3)—N2] are almost normal to one another, with a dihedral angle of 78.62 (9)°. The planes of the symmetry-related phthalimide moieties [N1/C1–C8 and N1^i^/C1^i^–C8^i^; symmetry code: (i) −*x*, *y*, −*z* + 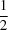] are inclined to one another by 73.53 (7)°.

## Supra­molecular features   

**Figure 2:**
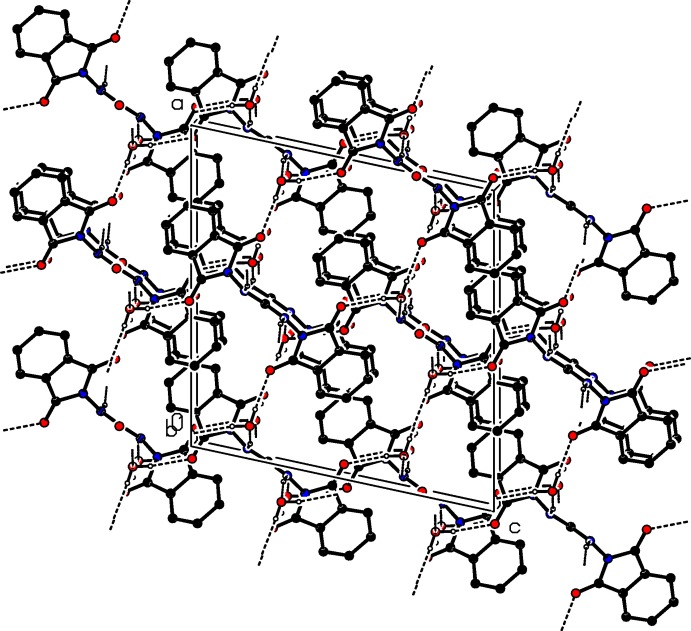
A view along the *b* axis of the crystal packing of the title compound. Hydrogen bonds are shown as dashed lines (see Table 1 ▶ for details. C-bound H atoms have been omitted for clarity.

**Figure 3:**
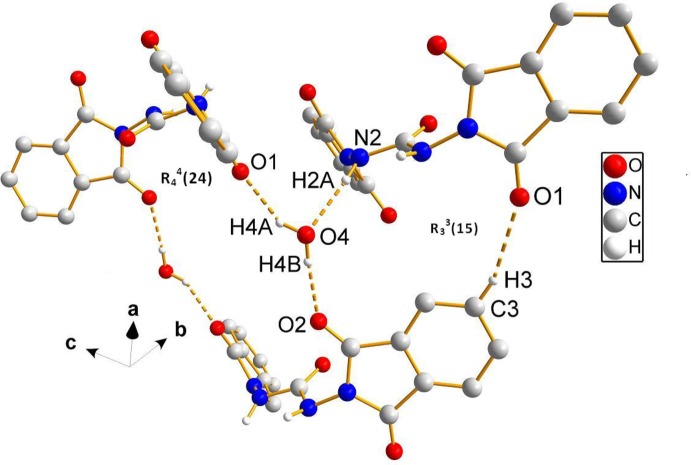
A view of the crystal packing of the title compound. The hydrogen bonds (dashed lines; see Table 1 ▶ for details) enclose 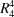
(24) and 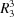
(15) ring motifs.

**Figure 4:**
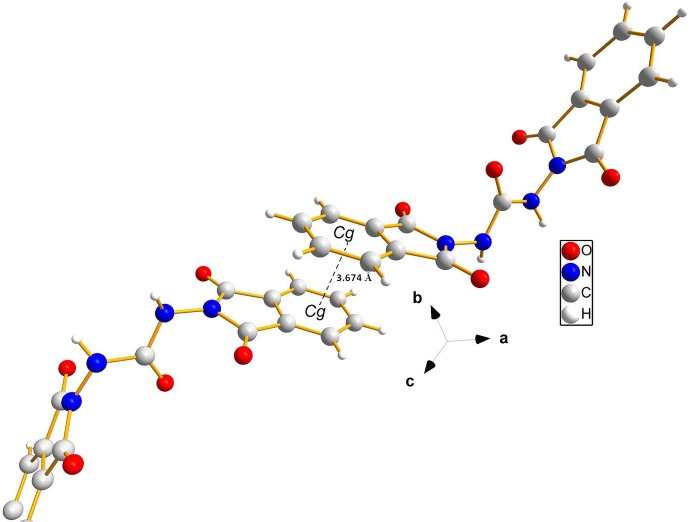
Two mol­ecules of the title compound showing the offset π–π inter­actions involving the benzene rings of neighbouring phthalimide moieties (dashed line).

In the crystal, mol­ecules are linked by N—H⋯O and O—H⋯O hydrogen bonds, forming a three-dimensional framework structure (Table 1 ▶ and Fig. 2 ▶). The solvent water mol­ecules, which occupy general positions, take part in the hydrogen-bonding network (Table 1 ▶ and Figs. 2 ▶ and 3 ▶). The O atom of the water mol­ecules, O4, is an acceptor of one H atom and simultaneously a donor of their two H atoms and enclose 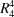
(24) and 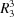
(15) ring motifs (Table 1 ▶ and Fig. 3 ▶). The crystal packing is reinforced by C—H⋯O hydrogen bonds, and slipped parallel π–π inter­actions (Fig. 4 ▶) involving benzene rings of neighbouring phthalimide moieties [*Cg*⋯*Cg* ^i^ = 3.6746 (15) Å; normal distance = 3.3931 (9) Å; slippage = 1.411 Å; *Cg* is the centroid of the C1–C6 ring; symmetry code: (i) −*x* + 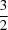, −*y* + 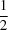, −*z* + 2].

## Synthesis and crystallization   

Carbohydrazide (0.5 g, 5.5 mmol) and phthalic anhydride (1.64 g, 11 mmol) were dissolved in dimethyl sulfoxide (15 ml) and refluxed for 6 h at 323 K. The solvent was removed under reduced pressure in a rotatory evaporator and the pale-yellow solid residue was washed with water and dried under vacuum. The product was recrystallized from water/ethanol (30:70 *v*/*v*) to give colourless prismatic crystals suitable for X-ray diffraction analysis (m.p. 491–493 K). ^1^H NMR (200 MHz, DMSO-*d*_6_, Me_4_Si): δ 9.25 (2H, N—H), 7.80 (8H, Ar). ^13^C NMR (50 MHz, DMSO-*d*_6_, Me_4_Si): δ 165.2 (C7, C8, C7′, C8′), 154.7 (C9), 135.0 (C5, C2, C5′, C2′), 129.4 (C1, C6, C1′, C6′), 123.5 (C3, C4, C3′, C4′). MS (FAB^+^): *m*/*z* (%) 349 (*M*—H, 25).

## Refinement details   

Crystal data, data collection and structure refinement details are summarized in Table 2 ▶. The NH group and water mol­ecule H atoms were located in a difference Fourier map and refined with distance restraints N—H = 0.86 (1) Å and O—H = 0.84 (1) Å, and with *U*_iso_(H) = 1.2*U*_eq_(N) and 1.5*U*_eq_(O). C-bound H atoms were positioned geometrically and constrained using a riding-model approximation, with C—H = 0.93 Å and*U*_iso_(H) = 1.2*U*_eq_(C).

## Supplementary Material

Crystal structure: contains datablock(s) I, New_Global_Publ_Block. DOI: 10.1107/S1600536814022144/su2791sup1.cif

Structure factors: contains datablock(s) I. DOI: 10.1107/S1600536814022144/su2791Isup2.hkl

CCDC reference: 1027988

Additional supporting information:  crystallographic information; 3D view; checkCIF report

## Figures and Tables

**Table 1:** Hydrogen-bond geometry (, )

*D*H*A*	*D*H	H*A*	*D* *A*	*D*H*A*
N2H2*A*O4^i^	0.87(2)	1.96(2)	2.811(3)	167(2)
O4H4*A*O1^ii^	0.85(1)	2.11(1)	2.891(3)	154(3)
O4H4*B*O2^iii^	0.85(2)	2.01(2)	2.850(3)	175(3)
C3H3O1^iv^	0.93	2.56	3.447(3)	160

Symmetry codes: (i) 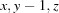; (ii) 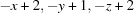; (iii) 
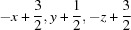
; (iv) 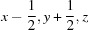.

**Table 2:** Experimental details

Crystal data	
Chemical formula	C_17_H_10_N_4_O_5_2H_2_O
*M*_r_	386.32
Crystal system, space group	Monoclinic, *C*2/*c*
Temperature (K)	293
*a*, *b*, *c* ()	15.268(3), 7.8053(16), 14.729(3)
()	102.097(3)
*V* (^3^)	1716.3(6)
*Z*	4
Radiation type	Mo *K*
(mm^1^)	0.12
Crystal size (mm)	0.40 0.32 0.23

Data collection	
Diffractometer	Bruker SMART CCD area detector
Absorption correction	Multi-scan (*SADABS*; Sheldrick, 2003 ▶)
*T*_min_, *T*_max_	0.954, 0.973
No. of measured, independent and observed [*I* > 2(*I*)] reflections	7038, 1529, 1414
*R*_int_	0.035
(sin /)_max_ (^1^)	0.597

Refinement	
*R*[*F* ^2^ > 2(*F* ^2^)], *wR*(*F* ^2^), *S*	0.052, 0.130, 1.12
No. of reflections	1529
No. of parameters	141
No. of restraints	4
H-atom treatment	H atoms treated by a mixture of independent and constrained refinement
_max_, _min_ (e ^3^)	0.37, 0.25

Computer programs: *SMART* and *SAINT-Plus* (Bruker, 2001 ▶), *SHELXS97*, *SHELXL97* and *SHELXTL-NT* (Sheldrick, 2008 ▶), *DIAMOND* (Brandenburg, 1997 ▶), *PLATON* (Spek, 2009 ▶) and *publCIF* (Westrip, 2010 ▶).

## supplementary crystallographic information

### Crystal data

**Table:**

C_17_H_10_N_4_O_5_·2H_2_O	*F*(000) = 800
*M**_r_* = 386.32	*D*_x_ = 1.495 Mg m^−^^3^
Monoclinic, *C*2/*c*	Mo *K*α radiation, λ = 0.71073 Å
Hall symbol: -C 2yc	Cell parameters from 5032 reflections
*a* = 15.268 (3) Å	θ = 2.6–28.1°
*b* = 7.8053 (16) Å	µ = 0.12 mm^−^^1^
*c* = 14.729 (3) Å	*T* = 293 K
β = 102.097 (3)°	Prism, colourless
*V* = 1716.3 (6) Å^3^	0.40 × 0.32 × 0.23 mm
*Z* = 4	

### Data collection

**Table:**

Bruker SMART CCD area-detector diffractometer	1529 independent reflections
Radiation source: fine-focus sealed tube	1414 reflections with *I* > 2σ(*I*)
Graphite monochromator	*R*_int_ = 0.035
Detector resolution: 8.3 pixels mm^-1^	θ_max_ = 25.1°, θ_min_ = 2.7°
phi and ω scans	*h* = −17→18
Absorption correction: multi-scan (*SADABS*; Sheldrick, 2003)	*k* = −9→9
*T*_min_ = 0.954, *T*_max_ = 0.973	*l* = −17→17
7038 measured reflections	

### Refinement

**Table:**

Refinement on *F*^2^	Primary atom site location: structure-invariant direct methods
Least-squares matrix: full	Secondary atom site location: difference Fourier map
*R*[*F*^2^ > 2σ(*F*^2^)] = 0.052	Hydrogen site location: inferred from neighbouring sites
*wR*(*F*^2^) = 0.130	H atoms treated by a mixture of independent and constrained refinement
*S* = 1.12	*w* = 1/[σ^2^(*F*_o_^2^) + (0.0524*P*)^2^ + 1.5592*P*] where *P* = (*F*_o_^2^ + 2*F*_c_^2^)/3
1529 reflections	(Δ/σ)_max_ = 0.001
141 parameters	Δρ_max_ = 0.37 e Å^−^^3^
4 restraints	Δρ_min_ = −0.25 e Å^−^^3^

### Special details

**Table:**

Geometry. All e.s.d.'s (except the e.s.d. in the dihedral angle between two l.s. planes) are estimated using the full covariance matrix. The cell e.s.d.'s are taken into account individually in the estimation of e.s.d.'s in distances, angles and torsion angles; correlations between e.s.d.'s in cell parameters are only used when they are defined by crystal symmetry. An approximate (isotropic) treatment of cell e.s.d.'s is used for estimating e.s.d.'s involving l.s. planes.
Refinement. Refinement of *F*^2^ against ALL reflections. The weighted *R*-factor *wR* and goodness of fit *S* are based on *F*^2^, conventional *R*-factors *R* are based on *F*, with *F* set to zero for negative *F*^2^. The threshold expression of *F*^2^ > σ(*F*^2^) is used only for calculating *R*-factors(gt) *etc*. and is not relevant to the choice of reflections for refinement. *R*-factors based on *F*^2^ are statistically about twice as large as those based on *F*, and *R*- factors based on ALL data will be even larger.

### Fractional atomic coordinates and isotropic or equivalent isotropic displacement parameters (Å^2^)

**Table:**

	*x*	*y*	*z*	*U*_iso_*/*U*_eq_	
O1	1.04237 (9)	0.1919 (2)	1.00070 (10)	0.0522 (4)	
O2	0.79568 (10)	0.2183 (3)	0.76586 (10)	0.0646 (5)	
O3	1.0000	0.3375 (3)	0.7500	0.0527 (6)	
O4	0.89221 (13)	0.7577 (3)	0.80226 (14)	0.0780 (6)	
N1	0.92631 (11)	0.1729 (2)	0.87283 (11)	0.0453 (5)	
N2	0.97120 (12)	0.0845 (2)	0.81541 (12)	0.0477 (5)	
C1	0.82495 (13)	0.3490 (3)	0.91942 (14)	0.0424 (5)	
C2	0.75114 (14)	0.4447 (3)	0.92811 (17)	0.0528 (6)	
H2	0.7014	0.4533	0.8795	0.063*	
C3	0.75383 (16)	0.5273 (3)	1.01160 (18)	0.0589 (6)	
H3	0.7050	0.5926	1.0193	0.071*	
C4	0.82743 (16)	0.5149 (3)	1.08370 (19)	0.0611 (6)	
H4	0.8270	0.5716	1.1392	0.073*	
C5	0.90231 (15)	0.4194 (3)	1.07526 (16)	0.0515 (6)	
C6	0.89956 (12)	0.3377 (2)	0.99210 (13)	0.0398 (5)	
C7	0.96745 (13)	0.2286 (3)	0.96152 (13)	0.0392 (5)	
C8	0.84162 (14)	0.2441 (3)	0.84167 (14)	0.0458 (5)	
C9	1.0000	0.1824 (4)	0.7500	0.0416 (7)	
H5	0.9532 (16)	0.412 (3)	1.1262 (17)	0.057 (6)*	
H2A	0.9521 (17)	−0.0195 (17)	0.8048 (18)	0.068*	
H4B	0.8366 (8)	0.741 (4)	0.784 (2)	0.085*	
H4A	0.905 (2)	0.740 (4)	0.8601 (8)	0.085*	

### Atomic displacement parameters (Å^2^)

**Table:**

	*U*^11^	*U*^22^	*U*^33^	*U*^12^	*U*^13^	*U*^23^
O1	0.0329 (8)	0.0684 (10)	0.0520 (9)	0.0035 (7)	0.0018 (6)	0.0030 (7)
O2	0.0456 (9)	0.1021 (14)	0.0418 (9)	−0.0006 (9)	−0.0005 (7)	0.0028 (8)
O3	0.0514 (13)	0.0556 (14)	0.0510 (12)	0.000	0.0105 (10)	0.000
O4	0.0571 (11)	0.0918 (14)	0.0762 (13)	−0.0158 (10)	−0.0063 (10)	0.0090 (11)
N1	0.0363 (9)	0.0633 (11)	0.0374 (9)	0.0055 (8)	0.0104 (7)	0.0008 (8)
N2	0.0495 (10)	0.0558 (11)	0.0418 (9)	−0.0005 (9)	0.0185 (8)	−0.0018 (8)
C1	0.0345 (10)	0.0471 (11)	0.0467 (11)	−0.0009 (9)	0.0108 (8)	0.0104 (9)
C2	0.0372 (11)	0.0571 (13)	0.0648 (14)	0.0039 (10)	0.0122 (10)	0.0169 (11)
C3	0.0480 (13)	0.0474 (13)	0.0889 (18)	0.0033 (10)	0.0314 (13)	0.0017 (12)
C4	0.0578 (15)	0.0566 (14)	0.0747 (15)	−0.0090 (11)	0.0273 (12)	−0.0181 (12)
C5	0.0443 (12)	0.0560 (13)	0.0548 (13)	−0.0088 (10)	0.0117 (10)	−0.0102 (10)
C6	0.0323 (10)	0.0427 (10)	0.0452 (11)	−0.0050 (8)	0.0100 (8)	0.0044 (8)
C7	0.0325 (10)	0.0469 (11)	0.0379 (10)	−0.0038 (8)	0.0069 (8)	0.0056 (8)
C8	0.0358 (11)	0.0636 (13)	0.0376 (11)	−0.0025 (9)	0.0070 (9)	0.0094 (9)
C9	0.0323 (14)	0.0539 (18)	0.0371 (14)	0.000	0.0039 (11)	0.000

### Geometric parameters (Å, º)

**Table:**

O1—C7	1.203 (2)	C1—C8	1.472 (3)
O2—C8	1.205 (2)	C2—C3	1.381 (3)
O3—C9	1.210 (4)	C2—H2	0.9300
O4—H4B	0.846 (10)	C3—C4	1.378 (3)
O4—H4A	0.844 (10)	C3—H3	0.9300
N1—N2	1.380 (2)	C4—C5	1.392 (3)
N1—C8	1.394 (3)	C4—H4	0.9300
N1—C7	1.395 (3)	C5—C6	1.374 (3)
N2—C9	1.373 (2)	C5—H5	0.96 (2)
N2—H2A	0.865 (10)	C6—C7	1.483 (3)
C1—C2	1.380 (3)	C9—N2^i^	1.373 (2)
C1—C6	1.393 (3)		
			
H4B—O4—H4A	108 (3)	C3—C4—H4	119.3
N2—N1—C8	122.91 (16)	C5—C4—H4	119.3
N2—N1—C7	123.07 (16)	C6—C5—C4	117.1 (2)
C8—N1—C7	112.88 (17)	C6—C5—H5	122.4 (14)
C9—N2—N1	115.14 (19)	C4—C5—H5	120.5 (14)
C9—N2—H2A	122.8 (18)	C5—C6—C1	121.5 (2)
N1—N2—H2A	112.8 (18)	C5—C6—C7	130.16 (19)
C2—C1—C6	121.0 (2)	C1—C6—C7	108.31 (17)
C2—C1—C8	130.59 (19)	O1—C7—N1	124.85 (19)
C6—C1—C8	108.40 (17)	O1—C7—C6	130.25 (19)
C1—C2—C3	117.6 (2)	N1—C7—C6	104.90 (16)
C1—C2—H2	121.2	O2—C8—N1	123.9 (2)
C3—C2—H2	121.2	O2—C8—C1	130.7 (2)
C4—C3—C2	121.4 (2)	N1—C8—C1	105.38 (16)
C4—C3—H3	119.3	O3—C9—N2^i^	123.86 (13)
C2—C3—H3	119.3	O3—C9—N2	123.86 (13)
C3—C4—C5	121.4 (2)	N2^i^—C9—N2	112.3 (3)
			
C8—N1—N2—C9	69.0 (2)	C8—N1—C7—C6	3.9 (2)
C7—N1—N2—C9	−97.9 (2)	C5—C6—C7—O1	−3.6 (4)
C6—C1—C2—C3	−0.5 (3)	C1—C6—C7—O1	176.4 (2)
C8—C1—C2—C3	178.3 (2)	C5—C6—C7—N1	176.7 (2)
C1—C2—C3—C4	0.0 (3)	C1—C6—C7—N1	−3.3 (2)
C2—C3—C4—C5	0.3 (4)	N2—N1—C8—O2	9.2 (3)
C3—C4—C5—C6	−0.2 (3)	C7—N1—C8—O2	177.3 (2)
C4—C5—C6—C1	−0.3 (3)	N2—N1—C8—C1	−171.03 (18)
C4—C5—C6—C7	179.7 (2)	C7—N1—C8—C1	−2.9 (2)
C2—C1—C6—C5	0.7 (3)	C2—C1—C8—O2	1.4 (4)
C8—C1—C6—C5	−178.36 (19)	C6—C1—C8—O2	−179.6 (2)
C2—C1—C6—C7	−179.32 (18)	C2—C1—C8—N1	−178.3 (2)
C8—C1—C6—C7	1.6 (2)	C6—C1—C8—N1	0.7 (2)
N2—N1—C7—O1	−7.7 (3)	N1—N2—C9—O3	11.43 (18)
C8—N1—C7—O1	−175.81 (19)	N1—N2—C9—N2^i^	−168.57 (18)
N2—N1—C7—C6	171.95 (17)		

Symmetry code: (i) −*x*+2, *y*, −*z*+3/2.

### Hydrogen-bond geometry (Å, º)

**Table:**

*D*—H···*A*	*D*—H	H···*A*	*D*···*A*	*D*—H···*A*
N2—H2*A*···O4^ii^	0.87 (2)	1.96 (2)	2.811 (3)	167 (2)
O4—H4*A*···O1^iii^	0.85 (1)	2.11 (1)	2.891 (3)	154 (3)
O4—H4*B*···O2^iv^	0.85 (2)	2.01 (2)	2.850 (3)	175 (3)
C3—H3···O1^v^	0.93	2.56	3.447 (3)	160

Symmetry codes: (ii) *x*, *y*−1, *z*; (iii) −*x*+2, −*y*+1, −*z*+2; (iv) −*x*+3/2, *y*+1/2, −*z*+3/2; (v) *x*−1/2, *y*+1/2, *z*.
